# ERGA-BGE reference genome of the Eurasian Woodcock ( *Scolopax rusticola*), a game bird species with isolated populations of conservation interest

**DOI:** 10.12688/openreseurope.23370.1

**Published:** 2026-04-13

**Authors:** Joan Ferrer Obiol, Diego Rubolini, Josephine R. Paris, Francesco Giannelli, David Gonçalves, Pedro Andrade, Beneharo Rodríguez, Alessandro Tedeschi, Michele Sorrenti, Chiara Natali, Marcella Sozzoni, Astrid Böhne, Rita Monteiro, Thomas Marcussen, Torsten H. Struck, Rebekah A. Oomen, Maria Angela Diroma, Claudio Ciofi, Caroline Howard, Mark Blaxter, Wellcome Sanger Institute Tree of Life Management: Samples and Laboratory Team, Aarushi Vaidya, Abitha Thomas, Adam Bates, Aleksandra Bliznina, Alex Makunin, Amit Vishwakumar, Amy Denton, Andy Griffiths, Anna Kovalevskaia, Arif Maulana, Benjamin Jackson, Cam Muyo, Caroline Howard, Charlotte Wright, Chloe Leech, Chris Laumer, Clare Cornwell, Claudia Weber, David Rowland, Ed Symons, Edel Sheerin, Elizabeth Sinclair, Ellen Cameron, Emma Teeling, Emmelien Vancaester, Erna King, Filipa Sampaio, Gene Myers, Graeme Oatley, Haddijatou Mbye, Halyna Yatsenko, Haoyu Niu, Ian Still, Isabelle Clayton-Lucey, Jack Monaghan, Jamie Davis, Jess Bernard, Jessica Thomas-Thorpe, Jessie Jay, Joana Meier, Jonah Walker, Juan Pablo Narváez Gomez, Kamil S Jaron, Keith Porter, Kerstin Howe, Lauma Ramona, Leah Bacon, Lewis Stevens, Liam Prestwood, Lora Downes, Lucy Kitchin, Luke Lythgoe, Maja Todorovic, Manuel Batista, Manuela Kieninger, Mara Lawniczak, Marcela Uliano-Silva, Maria Morra, Mark Blaxter, Martha Mulongo, Matthew Berriman, Max Brown, Molly Carter, Nancy Holroyd, Nicola Chapman, Paul Flicek, Priyanka Sethuk Raman, Radka Platte, Raquel Juliana Vionette do Amaral, Rebecca O'Brien, Richard Durbin, Robina Heathcote, Sam Ebdon, Sinead Calnan, Sophie Potter, Stephanie Fagan, Theodora Anderson, Victoria McKenna, Witek Morek, Yan Liang, Wellcome Sanger Institute Scientific Operations: Sequencing Operations, Abby Crackett, Abdulrahman Tuameh, Alexander Dove, Alexander Hatton, Alice Linsdell, Ana Monteiro, Barbora Pardubska, Ben Farr, Callum Murray, Carlos Jimenez Verdejo, Caroline Mitchell, Chris Henderson, Craig Corton, Danni Weldon, Elizabeth Easthope, Elliott Trigg, Emily Abraham, Emily Gallagher, Emma Dawson, Emma Memune Taluy, Esther Mellado Gomez, Filipa Sampaio, Francesco Iacoviello, Hannah Hanks, Harriet Johnson, Harriet Ninsiima, Henry Mallalieu, Hermione Blomfield-Smith, Ifeoluwapo Joshua, Iraad Bronner, Irene Fabiola Roman Maldonado, Jacqui Brown, James Du Preez, James Mack, James Uphill, James Watts, John Tushabe, Karen Oliver, Karolina Kujawa, Leanne Morrow, Lesley Shirley, Lucy Kitchin, Maariyah Rashid, Mary-Ann Santosh, Mia Franulovic, Michael A. Quail, Michelle Smith, Naomi R. Park, Neil Marriot, Nicholas Redshaw, Paul Heath, Ritoza Das, Robert Newell, Robin Moll, Sally Linsdell, Sarah Holmes, Scott Thurston, Shelly-Ann Coutts, Sophia Uvarova, Tavis Mason, Timi Adewumi, Tobi Ajenifuja, Tracey-jane Chillingworth, Tristram Bellerby, William Knight, Yousra Belattar, Zoe Goate, Chiara Bortoluzzi

**Affiliations:** 1 Dipartimento di Scienze e Politiche Ambientali, Università degli Studi di Milano, Milano, I-20133, Italy; 2 Department of Biology, Colorado State University, Fort Collins, Colorado, CO 80523, USA; 3 Department of Organismal Biology, Evolutionary Biology Centre, Uppsala University, Uppsala, Sweden; 4 CIBIO, Centro de Investigação em Biodiversidade e Recursos Genéticos, InBIO Laboratório Associado, Campus de Vairão, Universidade do Porto, Porto, Porto District, Portugal; 5 BIOPOLIS Program in Genomics, Biodiversity and Land Planning, CIBIO, Vairão, Portugal; 6 Departamento de Biologia, Faculdade de Ciências, Universidade do Porto, Porto, Portugal; 7 Buenavista del Norte, Canary Islands’ Ornithology and Natural History Group, Santa Cruz de Tenerife, 38480, Spain; 8 Associazione 'Amici di Scolopax', Via Roma 57, Mugnano del Cardinale (AV), I-83027, Italy; 9 Federazione Italiana della caccia, Via Garigliano 57, Ufficio Studi e Ricerche Faunistiche e Agro-Ambientali, Roma, I-00198, Italy; 10 Department of Biology, University of Florence, Sesto Fiorentino, Florence, Tuscany, I-50019, Italy; 11 Museum Koenig Bonn, Leibniz Institute for the Analysis of Biodiversity Change, Bonn, Germany; 12 University of Oslo Centre for Ecological and Evolutionary Synthesis, Oslo, Oslo, Norway; 13 University of Oslo, O. Box 1172, Blindern, Natural History Museum, Oslo, 0318, Norway; 14 Tjärnö Marine Laboratory, University of Gothenburg, Gothenburg, Sweden; 15 University of Agder, Centre for Coastal Research, Kristiansand, Norway; 16 Department of Biological Sciences, University of New Brunswick Saint John, Saint John, Canada; 17 Tree of Life, Wellcome Sanger Institute, Hinxton, England, CB10 1SA, UK; 18 Swiss Institute of Bioinformatics, Lausanne, Vaud, 1015, Switzerland

**Keywords:** Scolopax rusticola, genome assembly, European Reference Genome Atlas, Biodiversity Genomics Europe, Earth BioGenome Project, Aves, Scolopacidae, Eurasian Woodcock

## Abstract

The reference genome of the Eurasian Woodcock ( *Scolopax rusticola*) is an important resource to investigate population structure across the wide breeding range of this iconic game species and the conservation status of specific management units, such as the isolated Macaronesian populations. The genome sequence was assembled into 45 contiguous chromosomal pseudomolecules and 2 sex chromosomes (W and Z). This chromosome-level assembly encompasses 1.2 Gb, composed of 1,613 contigs and 935 scaffolds, with contig and scaffold N50 values of 5.9 Mb and 34.2 Mb, respectively.

## Introduction

**Figure 1.**
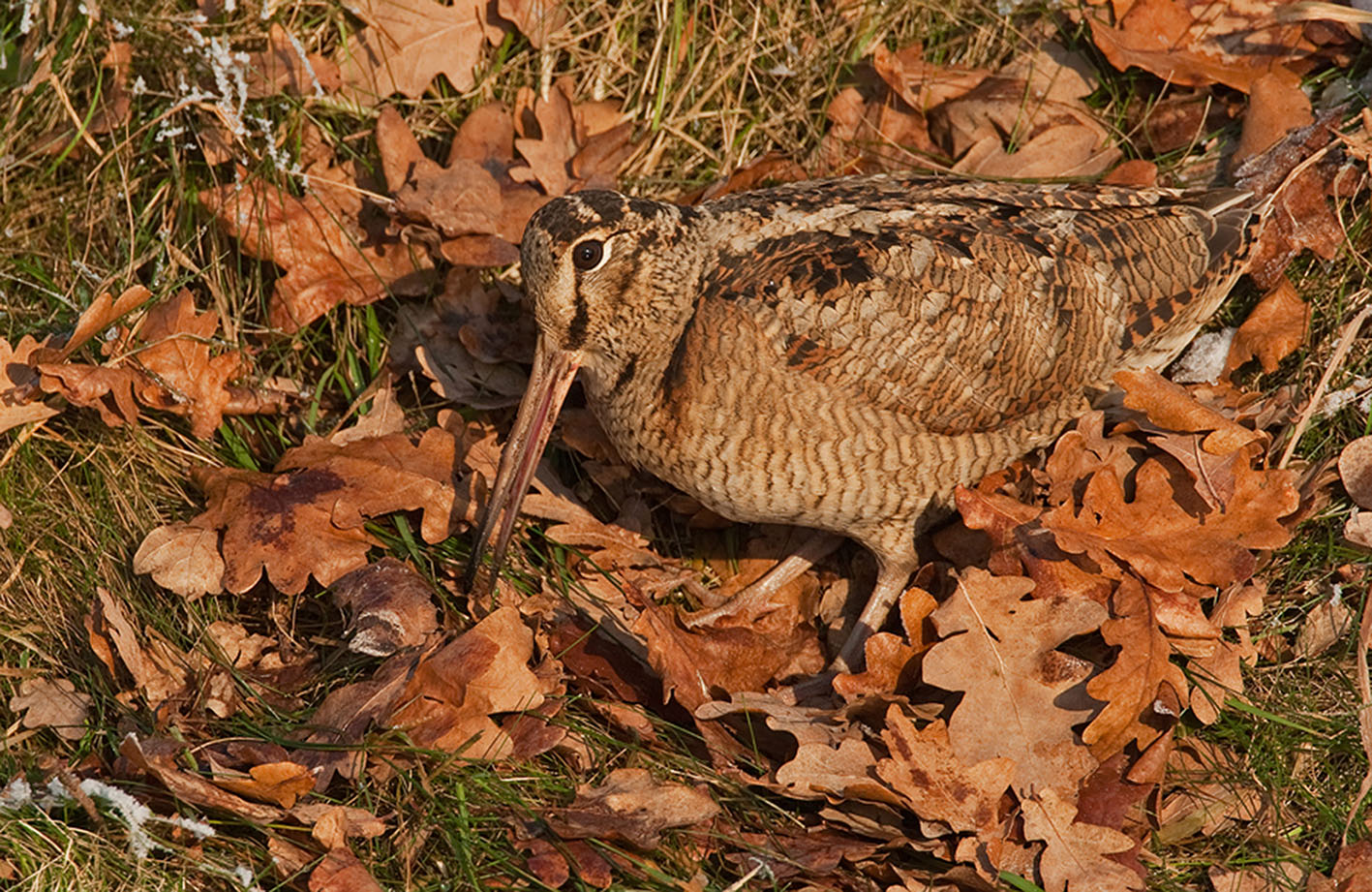
An adult Eurasian Woodcock displaying its typical cryptic plumage colouration. Image by Paul Cools, some rights reserved (CC BY-NC) ( https://www.inaturalist.org/photos/1935765).

The Eurasian Woodcock is a member of the *Scolopacidae* bird family ( Figure 1). Among members of its family, it stands out for its preference for terrestrial habitats, as it inhabits mature forests year-round, whereas most members of this family are strongly associated with aquatic habitats ( Cramp, 1998). The species is strictly nocturnal during most of its annual cycle ( Cramp, 1998; Duriez et al., 2004). The species has a broad breeding distribution range, spanning most of Eurasia ( Cramp, 1998). Northern breeding populations are highly migratory - birds from the north-eastern part of the breeding range can perform migratory flights exceeding 6000 km ( Arizaga et al., 2015; Tedeschi et al., 2020), spending the non-breeding period in southern Europe and the Mediterranean region (including north Africa).

The Eurasian Woodcock is currently classified as Least Concern by the IUCN, with a global population estimated at 10 to 16 million mature individuals ( BirdLife International, 2025). European breeding populations are considered ‘Decreasing’ ( BirdLife International, 2021). As an important game species, the Eurasian Woodcock is subjected to intense hunting pressure across most of its breeding and non-breeding range, including the small, isolated island populations in Macaronesia ( Andrade et al., 2022). An estimated 3–4 million individuals are hunted annually in Europe, most of which in France, Italy, and Greece ( Ferrand & Gossmann, 2001), and hunting pressure appears to be an important driver of survival and population dynamics ( Duriez et al., 2005; Prieto et al., 2019).

The species is a peculiar component of the taxonomic diversity of boreal and temperate forests, as it is the only Western Palearctic shorebird that relies exclusively on forest habitats throughout its annual cycle ( Cramp, 1998). Performing long-distance migrations, it contributes to the dispersal of many microorganisms and invertebrates (e.g., endoparasites) across Eurasia and Africa ( Paoletti et al., 2016), influencing their genetic diversity, dispersal, and population structure. It feeds mainly on soil invertebrates ( Cramp, 1998), thus contributing to regulating their population size and biomass.

The genome assembly presented here will serve as an important resource to investigate population structure in this widely distributed species and to deepen our understanding of the genetic and conservation status of isolated, genetically distinct, island populations in Macaronesia (see Andrade et al., 2022). The generation of this reference resource was coordinated by the European Reference Genome Atlas (ERGA) initiative’s Biodiversity Genomics Europe (BGE) project, supporting ERGA’s aims of promoting transnational cooperation to promote advances in the application of genomics technologies to protect and restore biodiversity ( Mazzoni et al., 2023).

## Materials & Methods

ERGA’s sequencing strategy to facilitate genome assembly and annotation includes Oxford Nanopore Technology (ONT) and/or Pacific Biosciences (PacBio) for long-read sequencing, along with Hi-C sequencing to reconstruct chromosomal conformation, Illumina Paired-End (PE) reads for polishing (i.e. recommended for ONT-only assemblies), and RNA sequencing for transcriptomic profilingn.

### Sample and sampling information

Joan Ferrer Obiol opportunistically collected tissues from one female specimen from Coppo, Ancona, Italy (43.51 N, 13.59 E) on 30 November 2023. At this location, woodcocks are routinely captured at night for scientific bird ringing by authorised and licensed expert operators, following standard and safe procedures commonly adopted for capturing this species (hand-held net mounted on a long pole and a flashlight; e.g., Duriez et al., 2004; Gossmann et al., 1988; Tedeschi et al., 2020). On that night, despite every precaution taken by ringers to avoid injuries and excessive stress to animals during capture, one captured individual accidentally died shortly after capture, not showing any external sign of damage, wound or haemorrhage. Joan Ferrer Obiol inferred that death likely resulted from capture myopathy, a fatal condition that can develop following capture events and has been frequently reported in wading birds, including woodcock, for reasons that remain unclear ( Breed et al., 2019; Rosenhagen, 2023). Muscle and brain tissue samples were thus opportunistically collected immediately after death, stored in dry ice shortly upon collection, and preserved at −80 °C until DNA extraction. Identification was performed by Joan Ferrer Obiol based on phenotypic appearance using standard references ( Cramp, 1998) and own solid ornithological expertise. The Eurasian woodcock is a very distinctive species, with no other similar taxa occurring in the sampling region ( Cramp, 1998). Sex was identified *postmortem* based on gonadal inspection. Authorization for temporary capture of woodcocks for scientific purposes was issued to the Department of Environmental Science and Policy of the University of Milan by the local authorities (Regione Marche, D.D. Settore Forestazione e Politiche Faunistico-Venatorie - SDA/AP-FM n. 701).

### Vouchering information

Frozen reference tissue material of muscle is available from the same individual at the Biobank of the Leibniz Institute for the Analysis of Biodiversity Change https://leibniz-lib.de/en/research/research-centres/zmb/bonn-location/biobank.html under the voucher ID ZFMK-TIS-89907.

### Genetic information

The estimated genome size, based on ancestral taxa, is 1.32 Gb. This is a diploid genome with a haploid number of 44 chromosomes (2n = 88). All information for this species was retrieved from Genomes on a Tree ( Challis et al., 2023).

### DNA/RNA processing

DNA was extracted from muscle tissue using the MagAttract HMW DNA Kit (Qiagen) following the manufacturer’s protocol. Nucleic acids yield was quantified in a Qubit fluorimeter using a Qubit 1X dsDNA HS Kit (Life Technologies), and DNA purity was assessed by comparing absorbance values at 260, 280 and 230 nm in a Tecan Infinite M200 Pro spectrophotometer using a NanoQuant plate for quantification of small volumes of nucleic acids (Tecan). Integrity of DNA was then assessed by pulsed-field gel electrophoresis. DNA was then preserved at −80 °C.

RNA was extracted using the Quick-RNA MicroPrep Kit (Zymo research) according to the manufacturer’s instructions. RNA was extracted from brain tissue. RNA quantification was performed using the Qubit RNA BR kit, and RNA integrity was assessed using a Bioanalyzer 2100 system RNA 6000 Nano Kit (Agilent). RNA was preserved at −80 °C until library preparation.

### Library preparation and sequencing

Whole DNA was sheared in a Megaruptor 2 DNA shearing system (Diagenode) using large fragment hydropores with a target mean fragment length of 20 kb. Fragment profile was assessed by capillary electrophoresis on a 5200 Fragment Analyzer system using an HS large fragment 50 kb kit (Agilent Technologies). Repair and A-tailing of DNA fragments and SMRTbell adapters ligation were performed according to the SMRTbell prep kit 3.0 protocol (Pacific Biosciences). A BluePippin automated pulsed-field gel electrophoresis system (Sage Science) was employed to remove fragments shorter than 10 kb. Primer annealing, polymerase binding and preparation of internal DNA control were performed using the Pacific Biosciences Sequel II binding kit and DNA internal control complex 3.2 according to the manufacturer’s protocol. The library was sequenced in HiFi mode in a Pacific Biosciences Sequel IIe platform using a Sequel II Sequencing plate 2.0 and two 8 M ZMW SMRT cells with a 30-hour movie time and 2 hours of pre-extension time for a target 25X genome coverage.

An RNA library was prepared using the Illumina Stranded mRNA library preparation and ligation kit, starting from 700 ng of total RNA and following the manufacturer’s protocol. RNA library was quantified in a Qubit Flex Fluorometer using an RNA BR assay, and library quality was checked by capillary electrophoresis in a 5200 Fragment Analyzer System. The library was then sequenced 2 × 150 paired end on an Illumina Novaseq 6000 platform to attain a 50X genome coverage. Hi-C data were generated from muscle tissue of bScoRus1 using the Arima-HiC v2 kit. For Hi-C library preparation, DNA was fragmented to a size of 400 to 600 bp using a Covaris E220 sonicator. The DNA was then enriched, barcoded, and amplified using the NEBNext Ultra II DNA Library Prep Kit (New England Biolabs) following the manufacturer’s instructions. The Hi-C sequencing was performed using paired-end sequencing with a read length of 150 bp on an Illumina NovaSeq X instrument. In total 33.5 Gb PacBio HiFi reads, 171 million Illumina RNA reads, and 80Gb HiC data were sequenced to generate the assembly.

### Genome assembly methods

The genome was assembled using the VGP Galaxy pipeline ( Larivière et al., 2024). Briefly, reads were preprocessed for quality and length using Cutadaptv Galaxy Version 5.0 + galaxy0, and initial contigs were assembled using Hifiasm Galaxy Version 0.24.0 + galaxy0, followed by scaffolding with YaHS Galaxy Version 1.2a.2 + galaxy2. Finally, assembled scaffolds were curated via manual inspection using Pretext v 1.0.0 with the Rapid Curation Toolkit ( https://gitlab.com/wtsi-grit/rapid-curation ) to remove any false joins and incorporate any sequences not automatically scaffolded into their respective locations in the chromosomal pseudomolecules (or super-scaffolds). We used the script MicroFinder.py ( https://github.com/sanger-tol/MicroFinder) to better reconstruct microchromosomes. Summary analysis of the released assembly (GCA_976944445.1) was performed using the ERGA-BGE Genome Report ASM Galaxy workflow ( https://doi.org/10.48546/workflowhub.workflow.1104.1).

## Results

### Genome assembly

**Figure 2.**
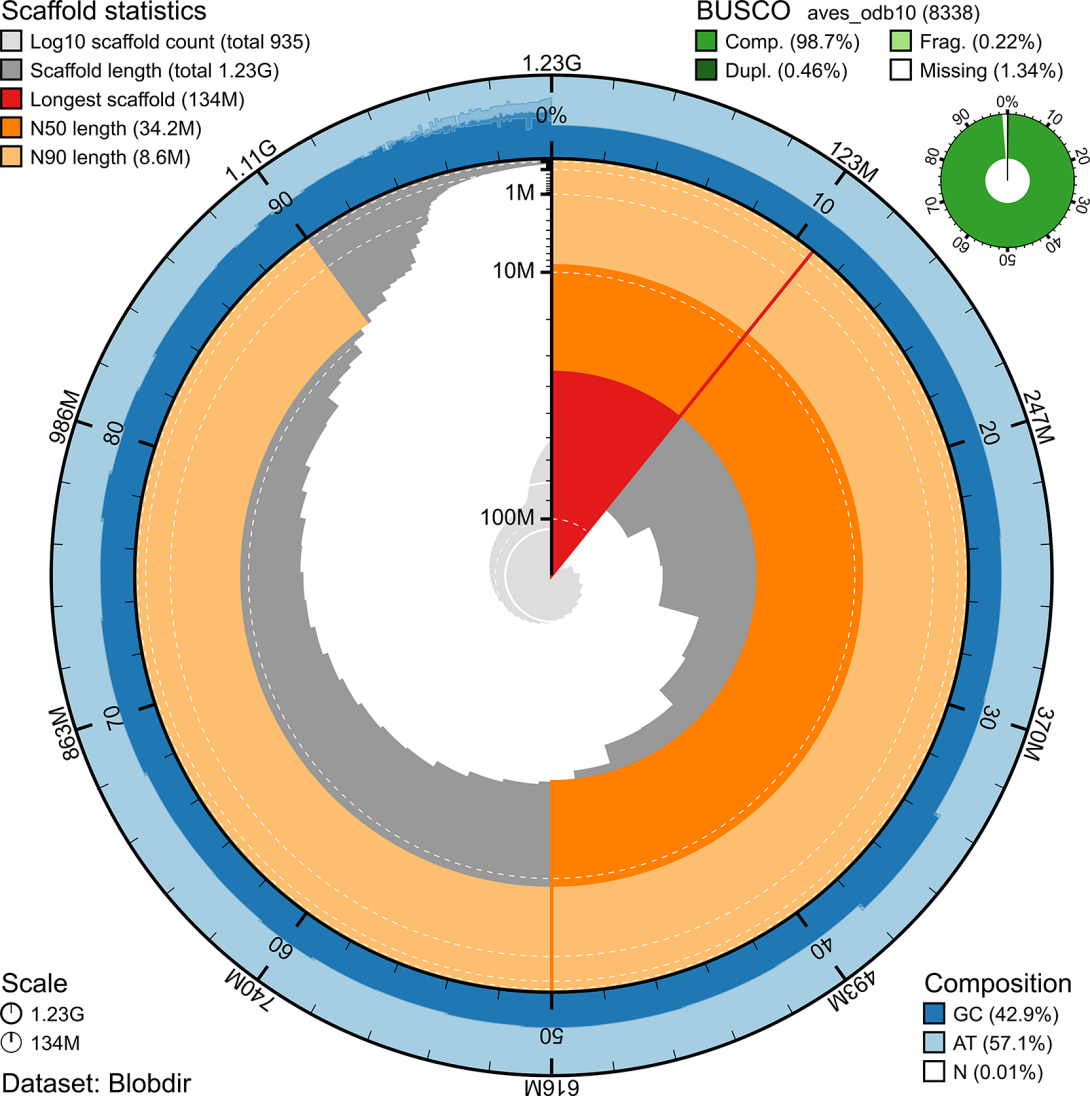
Snail plot summary of assembly statistics. The main plot is divided into 1,000 size-ordered bins around the circumference, with each bin representing 0.1% of the 1,232,635,287 bp assembly. The distribution of sequence lengths is shown in dark grey, with the plot radius scaled to the longest sequence present in the assembly (134 Mb, shown in red). Orange and pale-orange arcs show the scaffold N50 and N90 sequence lengths (34,162,297 and 8,602,126 bp), respectively. The pale grey spiral shows the cumulative sequence count on a log-scale, with white scale lines showing successive orders of magnitude. The blue and pale-blue area around the outside of the plot shows the distribution of GC, AT, and N percentages in the same bins as the inner plot. A summary of complete, fragmented, duplicated, and missing BUSCO genes found in the assembled genome from the Aves database (odb10) is shown in the top right.

**Figure 3.**
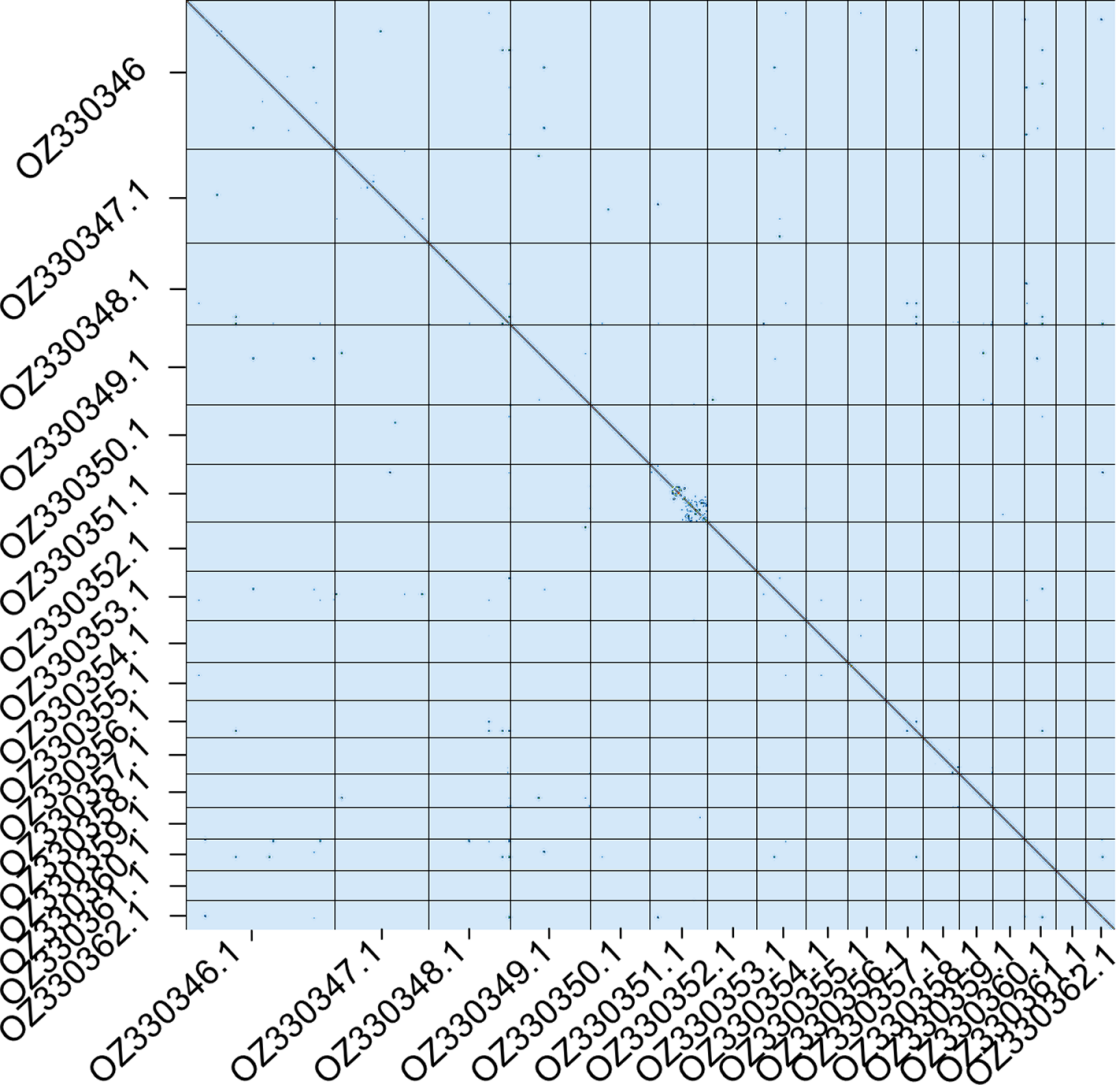
Hi-C contact map showing spatial interactions between regions of the genome. The diagonal corresponds to intra-chromosomal contacts, depicting chromosome boundaries. Chromosomes are shown on both x-axis and y-axis.

The genome assembly has a total length of 1,232,635,287 bp in 935 scaffolds ( Figures 2 & 3), with a GC content of 42.9%. The assembly has a contig N50 of 5,888,542 bp and L50 of 51, and a scaffold N50 of 34,162,297 bp and L50 of 10. The assembly has 678 gaps, totalling 135.6 kb in cumulative size. The single-copy gene content analysis using the Aves database (odb10) with BUSCO ( Manni et al., 2021) resulted in 98.7% completeness (98.2% single and 0.5% duplicated). 84.4% of reads k-mers were present in the assembly, and the assembly has a base accuracy Quality Value (QV) of 61.84 as calculated by Merqury ( Rhie et al., 2020).

## Author contributions

DR, JFO, JRP, DG, PA, BR, AT and MS coordinated the project within the framework of a broader collaboration involving the study of Eurasian woodcock population genomics; JFO and FG collected the species; JFO identified the species; JFO sampled and preserved biological material and provided metadata; AsB, RM, TM, THS, and RAO provided support in sampling, shipping of biological material, metadata collection, and management; CN extracted DNA, prepared libraries, and performed sequencing under the supervision of CC; WSOTOL Management and WSISO extracted DNA, prepared libraries, and performed sequencing under the supervision of CH; MS performed genome assembly and curation; MAD performed data curation; CB generated the analysis and report. All authors contributed to the writing, review, and editing of this genome note and read and approved the final version.

## Data Availability

*Scolopax rusticola* and the related genomic study were assigned to Tree of Life ID (ToLID) ‘bScoRus1’ and all sample, sequence, and assembly information are available under the umbrella BioProject PRJEB77651. The sample information is available at the following BioSample accessions: SAMEA115083842, SAMEA115083846, SAMEA115083847, and SAMEA115083848. The genome assembly is accessible from ENA under accession number GCA_976944445.1 and the annotated genome will be made available through the Ensembl website ( https://projects.ensembl.org/erga-bge/). Sequencing data produced as part of this project are available from ENA at the following accessions: ERX12721989, ERX12721990, ERX13270910, and ERX14241334. Documentation related to the genome assembly and curation can be found in the ERGA Assembly Report (EAR) document available at https://github.com/ERGA-consortium/EARs/tree/main/Assembly_Reports/Scolopax_rusticula/bScoRus1. Further details and data about the project are hosted on the ERGA portal at https://portal.erga-biodiversity.eu/data_portal/100826.
