# Supplementary material for: ERGA-BGE reference genome of the Eurasian Woodcock ( Scolopax rusticola), a game bird species with isolated populations of conservation interest
Source: Open Res Eur. 2026 Jul 20;6:111. Originally published 2026 Apr 13. [Version 2] doi: 10.12688/openreseurope.23370.2 (PMC13329356; doi:10.12688/openreseurope.23370.2)
Supplement: Peer review history

# Reviewer report: Reviewer response for version 2

**DOI:** 10.21956/openreseurope.26596.r77730

**Published:** 2026-07-22

**Authors:** Sifiso M Lukhele

**Affiliations:** 1 University of Cyprus, Nicosia, Cyprus; 2 Biological Sciences, University of Cyprus, Nicosia, Nicosia, Cyprus

The authors have addressed all my comments; therefore, I have no further comments to make.

Are sufficient details of methods and materials provided to allow replication by others?

Yes

Is the rationale for creating the dataset(s) clearly described?

Yes

Are the datasets clearly presented in a useable and accessible format?

Yes

Are the protocols appropriate and is the work technically sound?

Yes

Reviewer Expertise:

Molecular biology, evolutionary biology, behavioural ecology, ornithology, conservation biology, colouration biology.

I confirm that I have read this submission and believe that I have an appropriate level of expertise to confirm that it is of an acceptable scientific standard.

# Reviewer report: Reviewer response for version 1

**DOI:** 10.21956/openreseurope.25283.r75763

**Published:** 2026-07-02

**Authors:** Leilton Willians Luna

**Affiliations:** 1 Duke University, Durham, USA

This Data note describes the assembly of a reference genome for *Scolopax rusticola* (Eurasian Woodcock). Although this species is not globally threatened, the availability of this reference genome represents a valuable resource for assessing genetic diversity and population structure, particularly in potentially isolated populations such as those in Macaronesia.

 The methods employed by the authors follow current best practices for reference genome generation and assembly, and the resulting genome is of high quality. I have no major concerns regarding the methodology or analyses presented, and therefore I support the indexing of this data note.

Are sufficient details of methods and materials provided to allow replication by others?

Yes

Is the rationale for creating the dataset(s) clearly described?

Yes

Are the datasets clearly presented in a useable and accessible format?

Yes

Are the protocols appropriate and is the work technically sound?

Yes

Reviewer Expertise:

Biogeography, Evolution, Conservation, Molecular Ecology, Population genomics, Ornithology

I confirm that I have read this submission and believe that I have an appropriate level of expertise to confirm that it is of an acceptable scientific standard.

# Response

**Published:** 2026-07-09

**Authors:** Chiara Bortoluzzi

**Affiliations:** Swiss Institute of Bioinformatics, Lausanne, Vaud, Switzerland

We thank very much the reviewer for their positive feedback!

# Reviewer report: Reviewer response for version 1

**DOI:** 10.21956/openreseurope.25283.r74378

**Published:** 2026-06-16

**Authors:** Sifiso M Lukhele

**Affiliations:** 1 University of Cyprus, Nicosia, Cyprus; 2 Biological Sciences, University of Cyprus, Nicosia, Nicosia, Cyprus

**Title: ERGA-BGE reference genome of the Eurasian Woodcock (Scolopax rusticola), a game bird species with isolated populations of conservation interest**

****

 The paper reports the assembly and characterization of a high-quality reference genome for the Eurasian Woodcock, an important game bird with isolated populations of conservation concern. The assembly will support research into population structure, conservation status, and the management of both widespread and isolated populations, particularly in Macaronesia. 

 Though I find the paper well written and the genome assembly method clear, I have a few very minor comments.

 The sampling rationale is acceptable, but it would be helpful to explicitly state whether additional individuals from other populations are planned for sequencing to capture broader genetic diversity.

 Since the methods section is highly technical, with detailed references to specific kits, protocols utilized, and software versions, while this is valuable for reproducibility, a brief lay summary or diagram of the sequencing assembly workflow would aid readers who are novices in genomics. I also think that terms such as contig N50 and scaffold N50 should at least be defined.

Are sufficient details of methods and materials provided to allow replication by others?

Yes

Is the rationale for creating the dataset(s) clearly described?

Yes

Are the datasets clearly presented in a useable and accessible format?

Yes

Are the protocols appropriate and is the work technically sound?

Yes

Reviewer Expertise:

Molecular biology, evolutionary biology, behavioural ecology, ornithology, conservation biology, colouration biology.

I confirm that I have read this submission and believe that I have an appropriate level of expertise to confirm that it is of an acceptable scientific standard.

# Response

**Published:** 2026-07-09

**Authors:** Chiara Bortoluzzi

**Affiliations:** Swiss Institute of Bioinformatics, Lausanne, Vaud, Switzerland

We would like to thank the reviewer for their positive review. We have addressed the minor comments are follow: 

1. No additional individuals are planned for sequencing in the context of this genome report. The individual sample was chosen as representative of the species for the generation of a reference genome. We note, however, that some of the coauthors of this genome report have generated whole-genome sequencing data for 35 individuals from across the species distribution, including Macaronesia. Analysis of that dataset relies on the reference genome presented here and will form the basis of a separate manuscript, currently in preparation. 
2. We have now clarified in the genome report the meaning of both contig N50 and scaffold N50. 
